# Comparison of decomposition algorithms for identification of single motor units in ultrafast ultrasound image sequences of low force voluntary skeletal muscle contractions

**DOI:** 10.1186/s13104-022-06093-1

**Published:** 2022-06-15

**Authors:** Robin Rohlén, Jun Yu, Christer Grönlund

**Affiliations:** 1 Department of Radiation Sciences, Biomedical Engineering, Umeå University, 901 87 Umeå, Sweden; 2 Department of Mathematics and Mathematical Statistics, Umeå University, 901 87 Umeå, Sweden

**Keywords:** Ultrafast ultrasound, Concentric needle electromyography, Motor units, Decomposition algorithms, Blind source separation

## Abstract

**Objective:**

In this study, the aim was to compare the performance of four spatiotemporal decomposition algorithms (stICA, stJADE, stSOBI, and sPCA) and parameters for identifying single motor units in human skeletal muscle under voluntary isometric contractions in ultrafast ultrasound image sequences as an extension of a previous study. The performance was quantified using two measures: (1) the similarity of components’ temporal characteristics against gold standard needle electromyography recordings and (2) the agreement of detected sets of components between the different algorithms.

**Results:**

We found that out of these four algorithms, no algorithm significantly improved the motor unit identification success compared to stICA using spatial information, which was the best together with stSOBI using either spatial or temporal information. Moreover, there was a strong agreement of detected sets of components between the different algorithms. However, stJADE (using temporal information) provided with complementary successful detections. These results suggest that the choice of decomposition algorithm is not critical, but there may be a methodological improvement potential to detect more motor units.

**Supplementary Information:**

The online version contains supplementary material available at 10.1186/s13104-022-06093-1.

## Introduction

Blind source separation (BSS) separates a set of sources (e.g., hidden signals) from a set of mixtures of the sources (e.g., observed data) without information about the sources and the mixing process [1]. The goal of BSS is to jointly estimate the sources and the mixing process by only observing the mixture of the sources, which yields an ill-posed inverse problem. Many algorithms can solve a BSS problem [2–5], and they rely on different temporal, spatial, or spatiotemporal assumptions (different cost functions).

A typical BSS problem is identifying single motor units (MUs) from, e.g., multichannel data such as surface electromyography (EMG) [6]. The MU comprises a bundle of muscle fibres innervated by a motoneuron. Through neural activation, it electrically depolarizes the MU fibres (referred to as a firing instant) and gives rise to a muscle contraction [7, 8]. Studying the MUs’ function is essential in, e.g., diagnosing neuromuscular diseases [8], rehabilitation medicine [9], exercise physiology and sports sciences [10]. In previous work, our group applied a BSS algorithm called spatiotemporal independent component analysis (stICA) [3] to identify components in ultrafast ultrasound (UUS) image sequences [11, 12]. Using simulations, we showed that the method had high performance [11], but we could only identify about one-third of the active MUs in a validation study [12]. However, it’s still unknown whether this successful identification rate depends on that decomposition algorithm’s properties and cost function (also referred to as an error function that is pre-defined and minimized).

This study aimed to compare the performance of different spatiotemporal decomposition algorithms and parameters for identifying single MUs in human skeletal muscle under low force voluntary isometric contractions in UUS image sequences as an extension of a previous study [12]. The performance was quantified using two measures: (1) the similarity of components’ temporal characteristics against gold standard needle electromyography recordings and (2) the agreement of detected sets of components between the different algorithms. As a performance baseline, we also quantified performance without any decomposition.

## Main text

### Methods

#### Experimental procedure

**Fig. 1:**
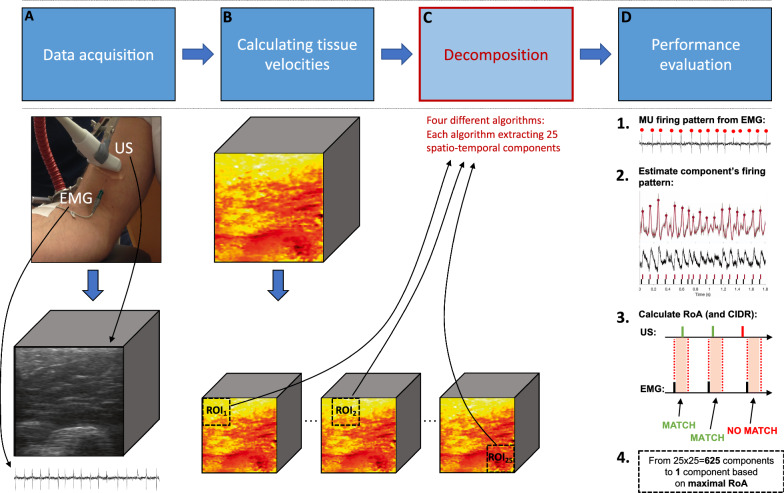
Framework for MU identification in ultrafast ultrasound (UUS) image sequences was composed of four stages. **A** The first stage; data acquisition. Collecting synchronized UUS and concentric needle electromyography (EMG) measurements on the biceps brachii under low force voluntary isometric contractions. **B **The second stage; calculating tissue velocities (based on the UUS radiofrequency signals). **C** The third stage; data decomposition. We inserted each region-of-interest (ROI, 25 in total) into four different decomposition algorithms (see Table 1) to extract 25 spatiotemporal components. **D** The fourth and final stage; post-processing. We selected one optimal component out of 625 (25 components in each of the 25 ROIs) based on its distance to the needle tip (< 10 mm) and maximal agreement to MU firing rate in terms of RoA. The selected components’ features are then compared between the different decomposition algorithms

We collected 64 synchronized measurements [12], from nine healthy subjects (27–45 years old, four men and five women), from the cross-section of biceps brachii (Fig. 1A). The synchronized measurements were collected using UUS (40 × 40 mm field of view, 2 kHz frame rate) and concentric needle-EMG (38 × 0.45 mm, 64 kHz sampling rate). The exclusion criteria were subjects with neuromuscular disease, blood disease, and subjects using blood-thinning drugs. The duration of each measurement was 2 s. Out of the 64 synchronized measurements, we extracted 91 firing patterns of single MUs from the 64 EMG datasets, where some datasets included multiple active MUs (Additional file 1: Table S2). A firing pattern is a sequence of firing instants. The subjects performed low force isometric elbow flexion as a physician inserted a needle electrode into the biceps brachii (about 1% of maximum voluntary contraction). An additional section file describes the data collection in more detail (Additional file 1: Data collection).

#### Framework for motor unit identification in ultrasound image sequences

Single MUs were identified using the framework described in Rohlén et al. [12], but we replaced the decomposition module. See Fig. 1. In short, we used a spatial sub-region of 20 × 20 mm as the region-of-interest (ROI) with jumps of 5 mm laterally and axially, resulting in 25 partially overlapping sub-regions (Fig. 1C). For each ROI, we reduced the data dimension using singular value decomposition. A decomposition algorithm is then applied to decompose each ROI into 25 spatiotemporal components (Fig. 1C), where we estimated the firing pattern for each component [12]. This procedure resulted in 25 × 25 = 625 components from each synchronized dataset and algorithm. From all these components, we selected one component per synchronized measurement (excluding components > 10 mm from the needle) based on the maximal rate of agreement (RoA) with the firing instants of the EMG-measured MU (Fig. 1D). For the RoA definition, see below.

#### Decomposition algorithms

The objective is to recover the latent components ***S*** from the observed data ***Y***. Here, we focused on the instantaneous linear model, ***Y = AS***, where ***Y = (Y****,…,****Y)*** is the observed data, ***S = (S****,…,****S)*** is the latent components, ***A*** is the unobserved mixing matrix, *m* and *n* are the numbers of pixels and latent components respectively. The objective is to transform the observed data ***Y*** using a linear transformation ***W = A***^**+**^$,$ which denotes the pseudo-inverse of ***A***. Thus, ***S = WY***. In this work, observed data ***Y*** is the UUS velocity data that has been vectorized from 3D (2D over time) to 2D.

We chose four algorithms to be evaluated with different parameters focusing on different spatiotemporal features (see Table 1 for an overview). For details of each algorithm, we refer to the corresponding articles. The selected decomposition algorithms are: sparse PCA (sPCA) [2], spatiotemporal independent component analysis (stICA) [3], spatiotemporal joint approximation diagonalization of eigenmatrices (stJADE) [4], and spatiotemporal second-order blind identification (stSOBI) [13]. sPCA has a parameter λ related to the number of non-zero pixels. In contrast, stICA, stJADE, and stSOBI have a weighting α-parameter favouring temporal or spatial separation [3].

**Table 1:** A summary of the selected decomposition algorithms and their parameters

Algorithm	Parameter (λ or α)	Domain	Description
sPCA	λ = 150	Spatial	Extension of principal component analysis (PCA) by sparse constraint, i.e., uses L_1_ penalty on the spatial loadings in the optimization procedure. λ denotes the number of non-zero pixels, a parameter equal to 150 and 250 corresponds to territories with 4.3 and 5.6 mm in diameter
	λ = 250	Spatial	
stICA	α = 0.0	Temporal	Separation by optimizing a joint entropy energy function based on mutual entropy and infomax with a kurtosis-based cost function. α = 0.8 has been used previously [11, 12], i.e., weighs 0.8 in spatial and 0.2 in temporal separation
	α = 0.8	Spatiotemporal	
	α = 1.0	Spatial	
stJADE	α = 0.0	Temporal	Joint diagonalization of fourth-order cumulant tensor in separation procedure. A low α weighs more on temporal separation
	α = 0.5	Spatiotemporal	
	α = 1.0	Spatial	
stSOBI	α = 0.0	Temporal	Autocovariance matrices (fixed number, 12) for joint diagonalization of a set of symmetrized multidimensional autocovariances [28, 29]. Similar to stJADE, a low α weighs more on temporal separation
	α = 0.5	Spatiotemporal	
	α = 1.0	Spatial	

α-parameter weighs spatial- and temporal separation, a λ-parameter relates to the number of non-zero pixels

*sPCA* sparse principal components, *stICA* spatiotemporal independent component analysis, *stJADE* spatiotemporal joint approximation diagonalization of eigenmatrices, and *stSOBI* spatiotemporal second-order blind identification

The most common general BSS algorithms are the stICA, stJADE, and stSOBI (or its special cases) and have been used in other BSS comparison studies [14–17]. For example, the Infomax-based approach [18] is a common algorithm identical to the maximum likelihood approach used here [19]. stSOBI [13] is a spatiotemporal extension to SOBI [5], which is an extension of AMUSE [20] and has been used in previous studies [21, 22]. We chose sPCA (with spatial cost function) to solve the BSS problem using a completely different penalty/optimization procedure than the other algorithms. Note that dimension reduction is included in sPCA. We anticipate that all these algorithms, together with their various parameters, should represent a broad spectrum of the instantaneous linear BSS space.

We calculated a baseline for the algorithms’ comparison; no decomposition (ND). As with the decomposition algorithms, we computed mean values in the overlapping ROIs of different sizes (20 × 20 mm, 10 × 10 mm, and 5 × 5 mm), i.e., ND20, ND10, and ND5. Thus, we computed $\frac{1}{m}\sum_{m\in {R}_{i}^{j}}{{\varvec{Y}}}_{m}$, where ${{\varvec{Y}}}_{m}$ is the observed data vector at pixel *m*, and ${R}_{i}^{j}$ denotes a set of indexes in the image where *j* is ND20, ND10, or ND5 and *i* is one of the overlapping ROIs. Note that *i* is of different lengths depending on *j* due to changing ROI sizes.

#### Performance evaluation

The firing pattern similarity between each component and MU was calculated using the RoA metric calculated as $\mathrm{RoA}=100\times {c}_{j}/({c}_{j}+{A}_{j}+{B}_{j})$, where ${c}_{j}$ is the number of firings of the *jth* firing pattern that was identified, ${A}_{j}$ and ${B}_{j}$ are the number of false identified firings and unmatched firings in the *jth* firing pattern, respectively. The tolerance between each firing of a MU and a component was set to 30 ms motivated by the unknown electromechanical delay [23] and potential noise of the decomposed component’s causing variation in each estimated component’s firings. We divided RoA of each algorithm into different groups of success, i.e., no-success ($0\%\le \mathrm{RoA}<50\%$), semi-success ($50\%\le \mathrm{RoA}<75\%$), and high-success ($75\%\le \mathrm{RoA}\le 100\%$). The motivation behind the thresholds is that 50% RoA is around the peak value for “no-decomposition,” and 75% RoA is around the average value in the successfully identified RoA group in [12].

To determine whether there was a pairwise difference in median RoA between stICA08 and the other decomposition algorithms, we tested the pairwise differences in median RoA using the two-sided Wilcoxon signed-rank test. The stICA08 was used as a reference algorithm because it has been used in previous studies [11, 12]. The *p*-values were adjusted for multiple testing based on the false discovery rate [24].

To quantify the agreement of detected sets of components between the different algorithms, we used the common id ratio (CIDR) metric that we define as the cardinality of intersection of sets divided by the minimum cardinality in each set where the identified stICA08 MU indices were used as a reference. The CIDR takes a value between 0 and 1, where CIDR = 1 means that we found the same set of components, and CIDR = 0 means that none of the detected components of the two methods is equal.

### Results

#### Performance evaluation: firing pattern

**Fig. 2:**
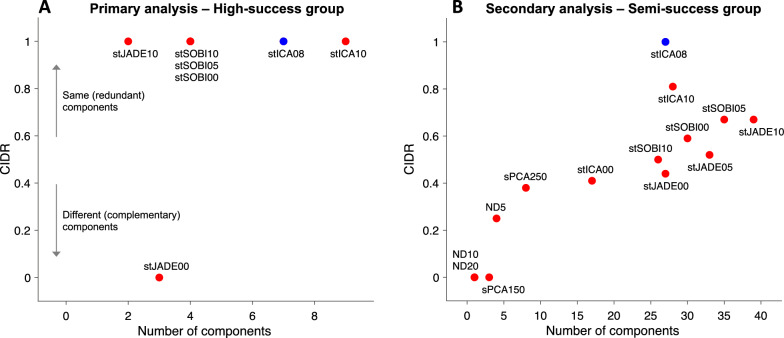
Performance evaluation of the decomposition algorithms (red points) with stICA08 (blue points) as the reference algorithm. The comparison between the algorithms’ performance is based on (1) firing pattern agreement between the components and the EMG reference (RoA), and (2) agreement between the different algorithms’ identified component sets (CIDR). The components’ RoA values were divided into groups; **A** high-success group (75% ≤ RoA ≤ 100%), and **B** semi-success group (50% ≤ RoA < 75%). Note that the number of components at the x-axis denotes each algorithm’s number of components within the pre-defined group (high-success or semi-success)

As a primary analysis, the high-success group is considered, as it relates to the successful one-third [12]. A secondary analysis relates to the semi-success group. Regarding the primary analysis, stICA08, stICA10, stJADE00, stJADE10, stSOBI00, stSOBI05, and stSOBI10 identified 2–9 components (2–10%) with RoA larger than 75% (Fig. 2A). There was no pairwise difference in median RoA between stICA08 and stICA10 (*p* = 0.21), stSOBI00 (*p* = 0.17), and stSOBI10 (*p* = 0.07). For all other algorithms, there was a statistically significant difference. ND and sPCA performed the worst, where 91–99% belonged to the no-success group.

Regarding the secondary analysis, there was no pairwise difference in median RoA between stICA08 and stICA10 (*p* = 0.26). For all other algorithms, there was a statistically significant difference. See the Additional file for examples and detailed descriptions (Additional file 1: Fig. S1–S3 and Table S1–S2).

#### Performance evaluation: agreement between detected sets of components

For the high-success group, 5 out of 6 algorithms had CIDR = 1.00, whereas stJADE00 had 0.00, meaning it complements stICA08 with a different set of components (Fig. 2A). None of the other algorithms did identify the same components as stJADE00 either (Additional file 1: Table S1). For the semi-success group, the CIDR range was 0.38–0.81 ($0.58\pm 0.14$, excluding ND and sPCA). stICA10, stJADE and stSOBI found about the same number of components (or more), and they centred at about CIDR = 0.6 (Fig. 2B), indicating improvement potential concerning stICA08. For the no-success group, the CIDR range was 0.79–0.98 ($0.85\pm 0.06$, excluding ND and sPCA).

### Discussion

We compared the performance of four different spatiotemporal decomposition algorithms and parameter settings for identifying single MUs in UUS image sequences of skeletal muscle low force voluntary isometric contractions. There are four main findings: (1) Out of these algorithms, no algorithm significantly improved the MU identification success compared to stICA08. (2) stICA with spatial approach and stSOBI with spatial or temporal approach had the best overall performance. (3) There was a strong agreement between different algorithms’ identified components. However, there were algorithms with complementary successful detections. (4) When no decomposition method was applied, 96–99% of the components belonged to the no-success group with an average agreement below 30%.

We found that stICA10 (spatial) and stSOBI00 (temporal) performed similarly in the high-success group. These two algorithms use entirely different cost functions. The former considers sparse territories, and the latter considers autocorrelated twitch trains, making sense because they should be sparse and autocorrelated due to the biological nature of the MU and twitch. Interestingly, stSOBI00 and stSOBI10 find the same high-RoA components, suggesting that temporal and spatial dependence are robust features for identification that could be adapted more explicitly using twitch-like a priori information. stICA00 (temporal) did not have any component that belonged to the high-success group suggesting that the twitch trains are not sparse, which also makes sense due to the non-stationary behaviour of twitches during an unfused tetanic contraction [25, 26]. Also, stJADE00 and stJADE10 found a few high-RoA units. A possible explanation why temporal stJADE00 managed to identify high-RoA components, which stICA00 could not, could be that the joint diagonalization approach is more robust against local minima and noise [4]. Also, stJADE00 complements the other algorithms with three new high-RoA components that were not identified by any other algorithm (Fig. 2A).

In conclusion, these findings suggest two things. (1) The choice of instantaneous decomposition algorithm is not critical for the present task. (2) There is an improvement potential to optimize the BSS cost function to detect more MUs in experimental image sequences of voluntary contracting skeletal muscles.

## Limitations

We assumed the firing pattern should be similar in EMG and UUS domains and the electromechanical delay was within the tolerance parameter in RoA [12], which is the only way to quantify successful identification in this case. We assumed an instantaneous linear mapping of the mixing matrix. However, a previous study suggests that the linear BSS algorithms may recover nonlinear mixed sources accurately if the input dimension is sufficiently higher than the source dimensionality [27].

## Supplementary Information

**Additional file 1.** A detailed description of the data collection. **Figure S1.** Pairwise firing pattern rate of agreement (RoA) differences for the three success groups. **Figure S2.** An example of components’ twitch trains for MU #30 regarding three algorithms’ (seven in total considering their different parameters). **Figure S3.** The individual rate of agreement (RoA) values for each motor unit (MU) and algorithm. **Table S1.** Performance evaluation of decomposition algorithms (in terms of RoA and CIDR). **Table S2.** The number of MUs extracted from the EMG data per contraction (91/64 = 1.4 active motor units per measurement/dataset).

## Data Availability

The data supporting this study’s findings are available on request from the corresponding author RR. The raw data are not publicly available because of the large file sizes.

## Abbreviations

UUS: Ultrafast ultrasound
MU: Motor unit
BSS: Blind source separation
stICA: Spatiotemporal independent component analysis
stJADE: Spatiotemporal joint approximation diagonalization of eigenmatrices
stSOBI: Spatiotemporal second-order blind identification
sPCA: Sparse principal component analysis
RoA: Rate of agreement
CIDR: Common id ratio
MUAP: Motor unit action potential
EMG: Electromyography
ND: No-decomposition
TVI: Tissue velocity images
ROI: Region of interest
