# Supplementary material for: Comparison of decomposition algorithms for identification of single motor units in ultrafast ultrasound image sequences of low force voluntary skeletal muscle contractions
Source: BMC Res Notes. 2022 Jun 15;15:207. doi: 10.1186/s13104-022-06093-1 (PMC9202224; doi:10.1186/s13104-022-06093-1)
Supplement: Supplementary file 1 — Additional file 1. A detailed description of the data collection. Figure S1. Pairwise firing pattern rate of agreement (RoA) differences for the three success groups. Figure S2. An example of components’ twitch trains for MU #30 regarding three algorithms’ (seven in total considering their different parameters). Figure S3. The individual rate of agreement (RoA) values for each motor unit (MU) and algorithm. Table S1. Performance evaluation of decomposition algorithms (in terms of RoA and CIDR). Table S2. The number of MUs extracted from the EMG data per contraction (91/64 = 1.4 active motor units per measurement/dataset). [file 13104_2022_6093_MOESM1_ESM.pdf]

Additional file 1:

Data collection

A physician inserted a standard clinical concentric needle into the biceps brachii muscle and was guided by the sound of active motor unit action potentials (MUAPs). The subject was instructed to generate a steady weak force by supinating the hand while the physician gave feedback based on the recorded MUAPs. The subject was asked to maintain this activation when the EMG signal showed a MUAP from one or a few motor units (MUs).

The EMG recordings were performed on a Cadwell Sierra Wave EMG system (Cadwell Laboratories Inc., Kennewick, WA, USA) with a 64 kHz sampling rate and a 38x0.45 mm concentric needle electrode (AMBU® Neuroline, DEN). We performed post-processing by removing a low-frequency synchronization signal effect (polynomial function) before identifying MUAPs by a method proposed by Stålberg et al. (1995) [1]. The MUAPs were manually double-checked, and a few superpositions were solved using manual editing.

An Ultrasonix SonixTouch (Ultrasonix Medical Corporation, Richmond, CA) with a 9 MHz L9-4 linear transducer and a 128 channel DAQ module was used to acquire two-second data with 40x40 mm field of view by plane wave transmit and parallel receive sequence resulting in an image rate of 2,000 images per second [2] (Fig. 1A in main paper). The radiofrequency data was reconstructed by sum-and-delay beamforming [3] and sampled at 40 MHz. The radiofrequency data was the basis for calculating axial tissue velocity images (TVI) [3] using a 2D autocorrelation approach [4] (Fig. 1B in the main paper). In the TVI-calculation, we used a sliding window of 10 ms and 1 mm maximal displacement in the depth direction for all channels between subsequent images. A band-pass (2-15 MHz with order 6) pre-processing the

radiofrequency data. The velocity data was processed by a high-pass filter (5 Hz) along the time dimension and a spatial 2-D median filtering (1x1 mm kernel) [5].

A customized synchronization procedure synchronized the EMG- and US systems. An optically isolated pushbutton switch was connected to a five mV DC in parallel on the EMG recording channel. A high-speed switch connected in series with the trigger signal from the main SonixTouch unit output to the scanline trigger input channel of the DAQ module. The master clock of the US was 50 MHz (and 64 kHz sample rate for the EMG).

Abbreviations

UUS: ultrafast ultrasound; MU: motor unit; BSS: blind source separation; stICA: spatiotemporal independent component analysis; stJADE: spatiotemporal joint approximation diagonalization of eigenmatrices; stSOBI: spatiotemporal second-order blind identification; sPCA: sparse principal component analysis; RoA: rate of agreement; CIDR: common id ration; MUAP: motor unit action potential; EMG: electromyography; ND: no-decomposition; TVI: tissue velocity images; ROI: region of interest.

Figures

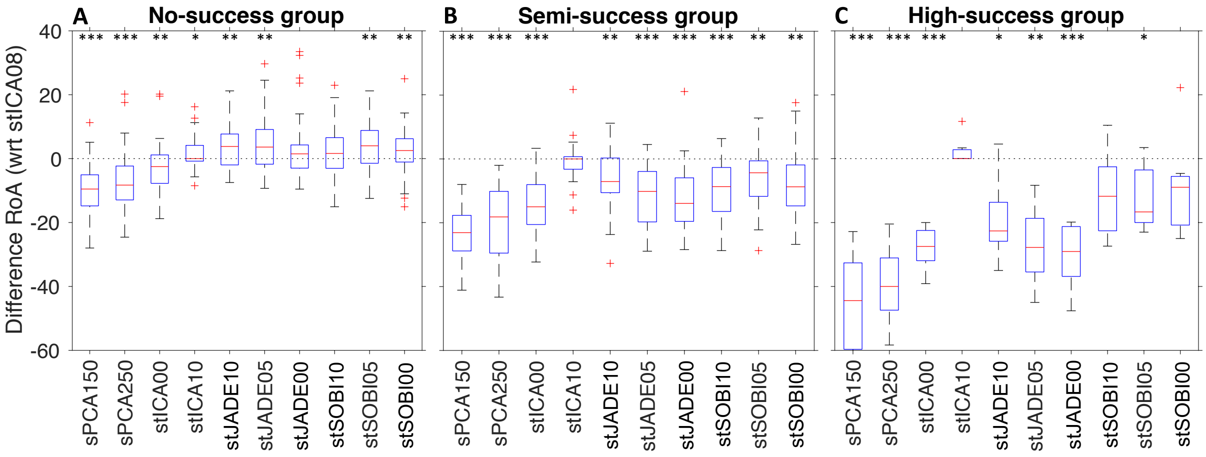

Fig. S1 Pairwise firing pattern rate of agreement (RoA) differences for the three success groups. **A** The no-success group (0% ≤ RoA < 50%): there was a statistical significance regarding the pairwise difference in median (with respect to stICA08) all algorithms except for stJADE00 and stSOBI10. **B** The semi-success group (50% ≤ RoA < 75%): There was no pairwise difference between in median RoA between stICA08 and stICA10 (p=0.26). For all other algorithms, there was a statistically significant difference in median RoA with respect to stICA08. **C** The high-success group (75% ≤ RoA ≤ 100%): There was no pairwise difference between in median RoA between stICA08 and stICA10 (p=0.21), stSOBI10 (p=0.07), and stSOBI00 (p=0.17). For all other algorithms, there was a statistically significant difference in median RoA with respect to stICA08. * p<0.05, ** p<0.01, *** p<0.001. The p-values were adjusted for multiple testing based on the false discovery rate method.

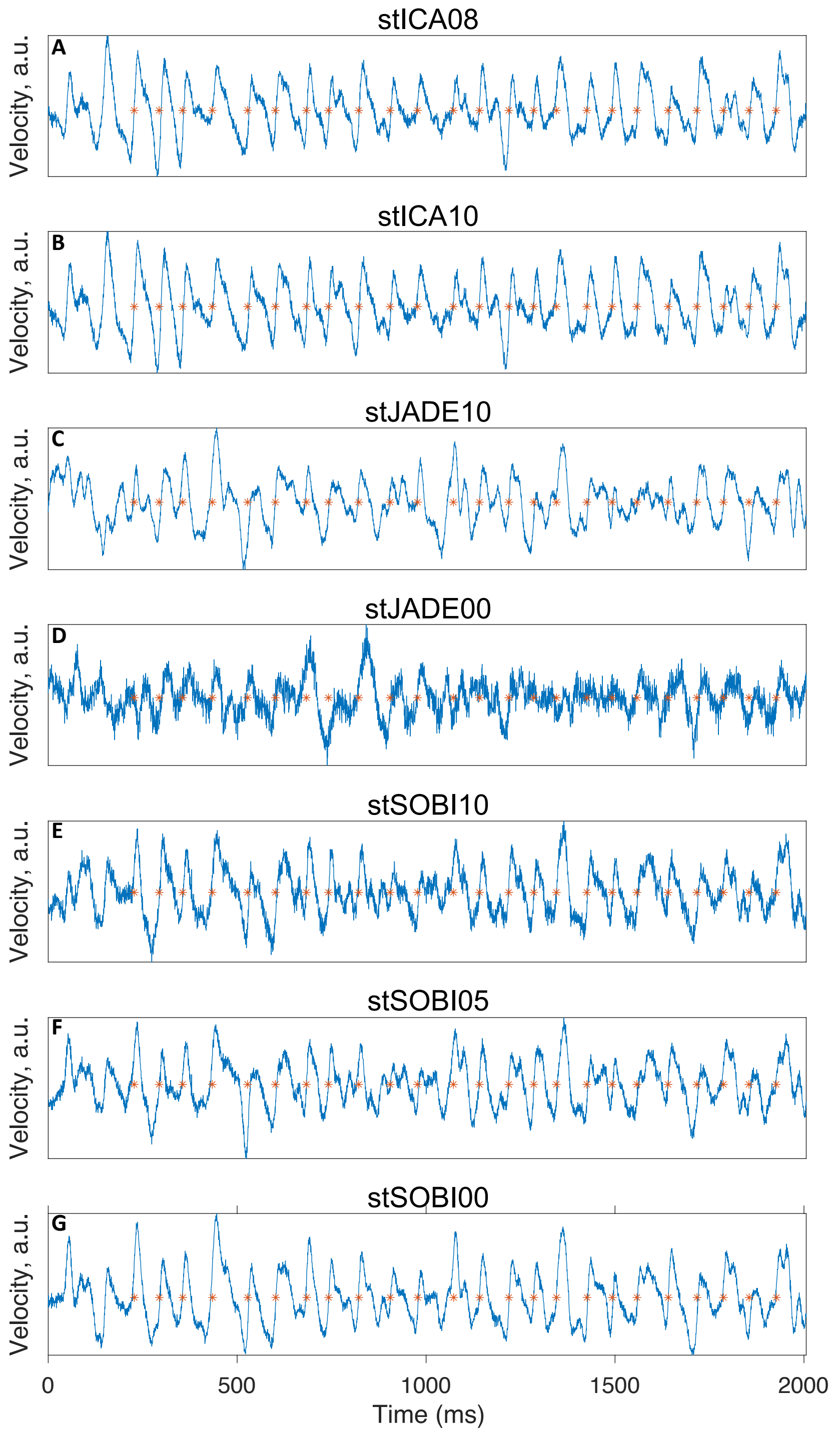

Fig. S2 An example of components' twitch trains for MU #30 regarding three algorithms' (seven in total considering their different parameters). These algorithms had identified components within the RoA high-success group: **A** stICA08, **B** stICA10, **C** stJADE10, **D** stJADE00, **E** stSOBI10, **F** stSOBI05, and **G** stSOBI00.

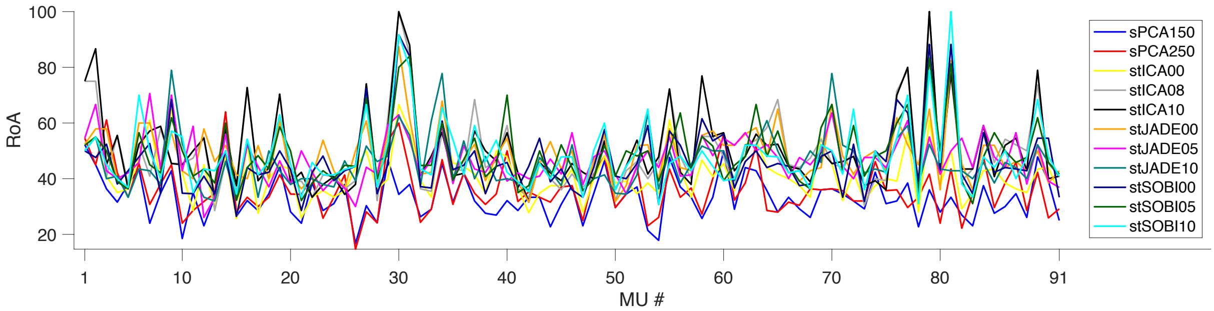

Fig. S3 The individual rate of agreement (RoA) values for each motor unit (MU) and algorithm. sPCA = sparse principal components, stICA = spatiotemporal independent component analysis, stJADE = spatiotemporal joint approximation diagonalization of eigenmatrices, and stSOBI = spatiotemporal second-order blind identification. 00, 05, 08 or 10 after each algorithm name refers to what parameter that was used.

Tables

Table S1 Performance evaluation of decomposition algorithms (in terms of RoA and CIDR).

	0 ≤ RoA < 50% No-success			50 ≤ RoA < 75% Semi-success			75 ≤ RoA ≤ 100% High-success			
	n (%)	mean ± std (min; max)	CIDR	*n* (%)	mean ± std (min; max)	CIDR	*n* (%)	mean ± std (min; max)	CIDR	MU #
**ND5**	87 (96)	30 ± 8 (17; 46)	0.95	4 (4)	56 ± 4 (52; 62)	0.25	0 (0)	-	-	-
**ND10**	90 (99)	27 ± 8 (11; 44)	0.98	1 (1)	59	0.00	0 (0)	-	-	-
**ND20**	90 (99)	23 ± 8 (6; 40)	0.98	1 (1)	55	0.00	0 (0)	-	-	-
**sPCA150**	88 (97)	33 ± 7 (17; 48)	0.96	3 (3)	53 ± 4 (50; 58)	0.00	0 (0)	-	-	-
**sPCA250**	83 (91)	34 ± 7 (15; 48)	0.95	8 (9)	56 ± 5 (50; 64)	0.38	0 (0)	-	-	-
**stICA00**	74 (81)	38 ± 6 (26; 48)	0.95	17 (19)	56 ± 5 (50; 67)	0.41	0 (0)	-	-	-
**stICA08**	57 (63)	41 ± 5 (29; 48)	**Ref.**	27 (30)	60 ± 8 (50; 74)	**Ref.**	7 (8)	84 ± 11 (75; 100)	**Ref.**	1, 2, 30, 31,77, 79, 81
**stICA10**	54 (59)	41 ± 4 (32; 48)	0.94	28 (31)	58 ± 7 (50; 74)	0.81	9 (10)	85 ± 10 (75; 100)	1.00	1, 2, 30, 31, 58, 77, 79, 81, 89
**stJADE00**	61 (67)	41 ± 4 (32; 48)	0.81	27 (30)	54 ± 4 (50; 64)	0.44	3 (3)	78 ± 1 (78; 79)	0.00	9, 34, 70
**stJADE05**	58 (64)	42 ± 5 (26; 48)	0.79	33 (36)	56 ± 6 (50; 71)	0.52	0 (0)	-	-	-
**stJADE10**	50 (55)	42 ± 4 (32; 48)	0.82	39 (43)	56 ± 5 (50; 68)	0.67	2 (2)	85 ± 4 (82; 88)	1.00	30, 81
**stSOBI00**	57 (63)	41 ± 5 (31; 48)	0.81	30 (33)	57 ± 7 (50; 70)	0.59	4 (4)	88 ± 10 (79; 100)	1.00	30, 31, 79, 81
**stSOBI05**	52 (57)	42 ± 4 (31; 48)	0.83	35 (39)	56 ± 6 (50; 70)	0.67	4 (4)	82 ± 2 (80; 84)	1.00	30, 31, 79, 81
**stSOBI10**	61 (67)	40 ± 5 (26; 48)	0.81	26 (29)	56 ± 6 (50; 72)	0.50	4 (4)	88 ± 3 (84; 92)	1.00	30, 31, 79, 81
**ALL+**	524	41 ± 5 (26; 48)	0.85 ± 0.06	262	57 ± 6 (50; 74)	0.58 ± 0.14	33	85 ± 8 (75; 100)	0.83 ± 0.41	-

stICA08 is the reference algorithm (Ref.). ND = no decomposition algorithm (only using mean in spatial and temporal dimension in each window), sPCA = sparse principal components, stICA = spatiotemporal independent component analysis, stJADE = spatiotemporal joint approximation diagonalization of eigenmatrices, and stSOBI = spatiotemporal second-order blind identification. 00, 05, 08 or 10 after each algorithm name refers to what parameter that was used (see Table 1). std = (sample) standard deviation. *n* = number of components. +Excluding ND and sPCA.

Table S2 The number of MUs extracted from the EMG data per contraction (91/64=1.4 active motor units per measurement/dataset).

# MUs	# Datasets	Total MUs
1	43	43
2	16	32
3	4	12
4	1	4
**Sum**	**64**	**91**

MUs = motor units. EMG = electromyography.

References

1. Stålberg E, Falck B, Sonoo M, Stålberg S, Åström M. Multi-MUP EMG analysis—a two year experience in daily clinical work. Electroencephalogr Clin Neurophysiol Mot Control. 1995;97:145–54.

2. Bercoff J. Ultrafast Ultrasound Imaging. In: Minin I V, Minin O V, editors. Ultrasound Imaging. Rijeka: IntechOpen; 2011.

3. Deffieux T, Gennisson JL, Tanter M, Fink M. Assessment of the mechanical properties of the musculoskeletal system using 2-D and 3-D very high frame rate ultrasound. IEEE Trans Ultrason Ferroelectr Freq Control. 2008;:2177–90.

4. Loupas T, Powers JT, Gill RW. An axial velocity estimator for ultrasound blood flow imaging, based on a full evaluation of the Doppler equation by means of a two-dimensional autocorrelation approach. IEEE Trans Ultrason Ferroelectr Freq Control. 1995;42:672–88.

5. Rohlen R, Stalberg E, Stoverud KH, Yu J, Gronlund C. A Method for Identification of Mechanical Response of Motor Units in Skeletal Muscle Voluntary Contractions Using Ultrafast Ultrasound Imaging - Simulations and Experimental Tests. IEEE Access. 2020;8:50299–311.
